# Comparative effectiveness and cost evaluation of Risankizumab and Adalimumab in the management of psoriasis: a real-world study in Saudi Arabia

**DOI:** 10.1186/s12962-023-00504-1

**Published:** 2023-12-09

**Authors:** Yazed AlRuthia, Almaha H. Alfakhri, Ibtisam Alharbi, Fadi Ali Alghamdi, Miteb A. Alanazi, Abdullah Abdulrahman Alrabiah, Anwar Abdulrazzag Alsouan, Abdulrahman Abdullah Alshaikh, Majed Ali Almasaoud

**Affiliations:** 1 Department of Clinical Pharmacy, College of Pharmacy, King Saud University, P.O. Box 2454, Riyadh, 11451 Saudi Arabia; 2 Pharmacoeconomics Research Unit, Department of Clinical Pharmacy, College of Pharmacy, King Saud University, P.O. Box 2454, Riyadh, 11451 Saudi Arabia; 3 Department of Pharmacy, King Saud University Medical City, P.O. Box 3145, Riyadh, 12372 Saudi Arabia; 4 Pharmacoeconomics Center, Department of Pharmacy, King Fahd Armed Forces Hospital, Jeddah, 23311 Saudi Arabia; 5 Department of Dermatology, King Fahd Armed Forces Hospital, Jeddah, 23311 Saudi Arabia; 6 College of Medicine, King Saud University, Riyadh, 12372 Saudi Arabia

**Keywords:** Psoriasis, Biologics, Effectiveness

## Abstract

**Background:**

Psoriasis, an immune-mediated chronic inflammatory disease primarily affecting skin and joints, has varying prevalence rates globally. It manifests in five types, with chronic plaque psoriasis being the most common. Treatment, which has no definitive cure, aims for complete resolution of skin symptoms and depends on disease extent, severity, and impact on patients’ lives. Biologics are an emerging treatment for psoriasis, targeting specific inflammatory pathways for potentially safer, more effective outcomes. However, these come with significant costs, necessitating more research to ensure value for money. This study aimed to compare the effectiveness of Risankizumab versus Adalimumab, the most commonly utilized biologic for managing psoriasis in Saudi Arabia.

**Methods:**

This study retrospectively compared the effectiveness and direct medical cost of Risankizumab and Adalimumab in treating chronic plaque psoriasis in adults from two Saudi Arabian healthcare centers. The Psoriasis Area and Severity Index (PASI) and body surface area (BSA) were used to assess treatment effectiveness, with patient data sourced from electronic medical records. Multiple regression analysis was performed to examine various factors affecting treatment outcomes. An economic evaluation was conducted to examine the cost-effectiveness of the two drugs, considering four scenarios with varying dosage patterns and costs. Analysis was performed from the perspective of public healthcare payers and considered all utilized health services.

**Results:**

The data for 70 patients were analyzed, with comparable baseline characteristics between groups. While Risankizumab led to a greater reduction in PASI scores and BSA affected, these results were not statistically significant. The annual treatment cost for Risankizumab was higher than Adalimumab. Various scenarios were studied, considering real acquisition costs, double dosing for Adalimumab, and the use of biosimilars. A scenario assuming double dosing for Adalimumab and a 40% discount for Risankizumab demonstrated both cost and efficacy advantages in 71.25% of cases.

**Conclusions:**

This study compared the effectiveness and cost of Risankizumab and Adalimumab for treating chronic plaque psoriasis in Saudi Arabian hospitals. Although Risankizumab showed a greater reduction in symptoms, the difference was not statistically significant. However, under certain scenarios, Risankizumab demonstrated cost and efficacy advantages. These findings may influence treatment decisions for psoriasis, but further research is needed.

## Background

Psoriasis is an immune-mediated chronic inflammatory disease with predominantly skin and joint involvement. Data indicate that prevalence rates can differ based on gender, geographical location, prevalence definition, study design, and case definition. Psoriasis can begin at any age, but it primarily presents in two peak age brackets: 30 to 39 and 50 to 69. These variations underscore the complexity of the disease and the influence of diverse environmental and genetic factors [1, 2]. The global prevalence of psoriasis varies considerably across all age groups (0.09–5.1%) and among adults (0.51–11.43%) [2]. Over the past two decades, there has been a notable increase in new psoriasis cases, rising from 92 to 99 per 10,000 [3]. In Saudi Arabia, there is limited data available. However, studies conducted in 2004 and 2005 reported a 1.5% and 3.4% prevalence in the south-western and eastern regions, respectively [4]. Furthermore, a retrospective study in a military hospital in Riyadh revealed 2.47% of 58,450 dermatology cases as psoriasis during 2001–2005 [5].

Psoriasis is associated with numerous comorbidities, including arthritis, depression, inflammatory bowel disease, and cardiovascular diseases [6]. Although its cause remains elusive, psoriasis is acknowledged as multifactorial, with dysregulated inflammatory responses and genetic links [7]. The disease manifests in five types—plaque psoriasis, guttate or eruptive psoriasis, inverse psoriasis, pustular psoriasis, and erythrodermic psoriasis [8]. The most prevalent form is chronic plaque psoriasis, affecting 80–90% of patients [7]. Besides skin, psoriasis can also trigger inflammatory arthritis, known as psoriatic arthritis, affecting the spine and other joints [8].

The diagnosis of psoriasis is primarily clinical and the severity is generally categorized as mild to moderate, and moderate to severe. Objective measures, such as the evaluation of the affected body surface area (BSA), the Psoriasis Area Severity Index (PASI), or a selection of Physician Global Assessment (PGA) methods, are employed to establish this in both clinical settings and research trials. Furthermore, the Dermatology Life Quality Index (DLQI) might be utilized to assess the influence of psoriasis on a patient’s quality of life [9, 10]. The PASI is a widely used instrument to assess and grade the severity and extent of psoriasis and response to treatment. The term PASI 75 denotes a 75% decrease from the initial PASI score, serving as a standard measure in numerous psoriasis clinical trials and the effectiveness criterion for novel treatments endorsed by the U.S. Food and Drug Administration (FDA). PASI 90 refers to a 90% reduction from baseline, which indicates a better improvement from PASI 75. Newer biologic agents for the treatment of moderate to severe psoriasis have achieved endpoints such as PASI 90 and PASI 100 in clinical trials, drawing attention to new treatment options that are highly desirable [11, 12].

There is no cure for psoriasis and treatment is mostly based on managing acute symptoms. The ultimate goal of any psoriasis treatment is to achieve complete resolution of skin disease with a well-tolerated treatment regimen. At least a 50% reduction in the baseline PASI is considered the minimum requirement for the efficacy of any therapy, if this is not achieved, treatment should be modified [10]. Psoriasis treatment regimens should be tailored to individual patients based on their disease extent and severity, response to previous regimens, and its impact on their quality of life. The existence of associated conditions, for instance, psoriatic arthritis, plays a crucial role in choosing the appropriate therapy [13, 14].

Therefore, various treatment regimens have been outlined for psoriasis, including topical agents, phototherapy, systemic and biological treatments. Mild to moderate psoriasis can be treated with topical agents such as glucocorticoids and vitamin D analogues, which can be combined with phototherapy. Systemic treatment is frequently necessary for managing moderate to severe cases of psoriasis [14]. Systemic therapies include biologic and nonbiologic treatments such as phototherapy and older systemic agents. Candidates for systemic therapy are patients who meet at least one of the following criteria: (1) BSA > 10%, (2) Disease involving areas affecting more impactful sites such as the face, palms, soles, genitalia, scalp, or nails, (3) Failure of topical therapy [9].

An improved understanding of the pathogenesis of psoriasis has led to the introduction of promising targeted biological therapies. Biologic agents used in the treatment of psoriasis include the anti-tumor necrosis factor (TNF) agents Adalimumab, Etanercept, Infliximab, and Certolizumab pegol; the anti-interleukin (IL)-12/IL-23 antibody Ustekinumab; the anti-IL-17 antibodies Secukinumab and Ixekizumab; the anti-IL-17 receptor antibody Brodalumab; and the anti-IL-23 antibodies Guselkumab, Tildrakizumab, and Risankizumab [15].

Psoriasis not only leads to physical impairment but also has a psychosocial and economic burden as it requires lifelong care [7]. It presents a significantly high economic burden that is comparable with other costly conditions such as pancreatic cancer, melanoma, prostate cancer, and asthma. Despite the currently available treatment options, psoriasis is associated with high costs in many countries, and the total cost is even higher when factoring the comorbidities associated with it. The incidence of psoriasis is increasing, indicating the need for treatments that offer good value for money [16]. A systematic review analyzed the cost of psoriasis and psoriatic arthritis in five European countries reported that total annual cost per patient ranged from US $2,077 To US $13,132 PPP for psoriasis, and from US $10,924 To US $17,050 PPP for psoriatic arthritis. The advent of biologics was associated with a three to five times surge in direct expenses, resulting in an overall rise in total costs [17].

Data from clinical trials suggest that various biologics are effective in treating psoriasis at 12 weeks. However, these outcomes may not fully represent real-world scenarios where combination therapies or extended treatment durations may be required [18]. Biologics such as TNFa inhibitors have shown to be effective in treating psoriasis, as validated by numerous randomized controlled trials (RCTs). However, their nonspecific mode of action and associated global immunosuppression are considered limitations to their use.

In contrast, IL inhibitors, which selectively target the inflammatory pathways linked to psoriasis, show greater specificity, potentially offering enhanced efficacy and safety [19]. Clinical trials have demonstrated remarkable improvements and faster onset of action with IL inhibitors compared to TNFa inhibitors [20]. Despite these promising results, IL inhibitors are relatively understudied, and robust data supporting their use is still lacking [21],

A network meta-analysis (NMA), that identified RCTs presenting data on direct comparisons of all biologics, reported that using their methodology, most biologics cluster together for short-term efficacy and tolerability, and no single agent was identified as ‘best’. The researchers determined that these findings should be considered in light of long-term efficiency, effectiveness data, safety measures, dosage instructions, and the cost of acquiring the medication while making therapeutic choices [22].

In another NMA that compared the short-term efficacy and safety of IL-23 targeted drugs in treating moderate to severe psoriasis, Risankizumab was found to be the most effective, and its risk of adverse events was not significantly different from placebo. However, more research data are needed in the cost-effectiveness field to evaluate which drug strikes the most favorable balance among efficacy, safety, and cost of access [23]. Risankizumab (Skyrizi®), is the most recently approved biologic for the treatment of adult psoriasis, it received its global approval in March 2019 in Japan. Phase 3 clinical trials of Risankizumab compared to placebo, and other biologics including Adalimumab, Ustekinumab, and Secukinumab showed superior efficacy in patients with moderate to severe plaque psoriasis by relative improvements from baseline in the PASI score [24–26]. However, the quality of evidence is not high [27, 28].

The performance of biologics in real-world scenarios may differ from RCTs since clinical trials often exclude certain patient groups, such as the elderly and those with multiple comorbidities. Consequently, there is a gap in real-life data on newer biological and biosimilar agents for moderate to severe psoriasis [29]. Comparative effectiveness studies are crucial in guiding healthcare providers and patients in making informed therapeutic decisions [20, 30].

Adalimumab and Risankizumab are commonly utilized biologics for the management of plaque psoriasis in Saudi Arabia with Risankizumab viewed more favorably than Adalimumab based on a published consensus statement by a group of Saudi experts [31]. However, the incremental clinical effectiveness of Risankizumab versus Adalimumab has not been investigated using real-world data despite the higher acquisition cost of Risankizumab. Therefore, this study was designed to compare the effectiveness of Risankizumab with Adalimumab in managing psoriasis among patients from various tertiary hospitals in Saudi Arabia. Moreover, we investigated the acquisition cost, incremental cost, number of visits, and patient hospitalization rates. The results should contribute to our understanding of the value of these biologics in managing psoriasis, providing crucial information for clinicians and policymakers.

## Methods

### Study design and study participants

This was a two-center, retrospective observational cohort study, focusing on adult patients (18 years and above) diagnosed with chronic plaque psoriasis for at least 12 months. The study population was selected from two tertiary healthcare facilities in Riyadh and Jeddah, Saudi Arabia. Our criteria included patients who had received either Adalimumab or Risankizumab treatment for at least three months. Those with a treatment duration of less than three months or having any malignancies or active infections were excluded. Moreover, patients with missing BSA or PASI scores observations were excluded. The study was conducted from the perspecctive of Saudi Arabia’s public healthcare payers.

### Data collection and study variables

The PASI was used to assess the effectiveness of Risankizumab versus Adalimumab among chronic plaque psoriasis patients. PASI is a valid measurement of the patient’s disease severity, such as discoloration, thickness, scaling, and the area affected by these plaques [32]. It generates a score ranging from 0 (no disease) to 72 (most severe form of disease), and has been widely used in clinical practice to assess disease progression and the effectiveness of treatment for different types of psoriasis (i.e., plaque psoriasis, Guttate psoriasis, inverse psoriasis, pustular psoriasis, erythrodermic psoriasis, nail psoriasis, and psoriatic arthritis) [32, 33]. PASI 75 is defined as ≥ 75% reduction in PASI scores from baseline. It is associated with significant improvement in psychological well-being and quality of life (QoL) after 12 weeks of treatment with monoclonal antibodies (mAbs), such as Adalimumab and Risankizumab, with no clinically significant difference in QoL when compared to PASI 90 after 12 and 24 weeks of treatment [11]. Three medical interns were involved in reviewing the medical records of patients with chronic plaque psoriasis and collecting relevant variables which included PASI score and BSA at baseline and last follow-up visit (i.e., three months at least), gender, age, duration of illness, duration of therapy, weight and height, and comorbidities (e.g., hypertension, dyslipidemia, asthma, cardiovascular disease). Micro-costing was employed to record all health services utilized during the follow-up period, including lab tests, imaging studies, hospitalization, emergency department visits, outpatient clinic visits, and nursing and physician fees. Data on the cost of different health services were retrieved from the Saudi Ministry of Health cost center. The data for 2020 and 2021 were collected, and the data collection started on December 21st 2021 and ended on September 7th 2022.

### Descriptive statistics and multiple regressions

The minimum sample size was determined to be 43 patients, calculated using an effect size of Cohen’s f2 = 0.15, α = 0.05, β = 0.2, power of 80%, and up to 6 predictor variables for multiple linear regression. Patients’ baseline characteristics were presented using means, standard deviations, frequencies, and percentages. Student’s t-test, Chi-square, Fisher’s exact tests were conducted, as appropriate, to compare the baseline characteristics of the patients on Risankizumab versus their counterparts on Adalimumab. Paired t-test was conducted to examine the difference in PASI scores and BSA (%) at baseline and last follow-up visit. Univariate regression analysis was conducted to examine the relationship between baseline and follow-up PASI scores. Furthermore, regression analyses were conducted to examine the relationship between baseline and follow-up PASI scores and the odds of achieving PASI 75 after at least three months of treatment with Risankizumab versus their counterparts treated with Adalimumab controlling for age, gender, duration of illness, duration of treatment, and number of comorbidities. Moreover, multiple linear regression was conducted to examine the impact of Risankizumab versus Adalimumab on the affected BSA (%) controlling for age, gender, duration of illness, duration of treatment, and number of comorbidities.

### Comparative economic evaluation of Risankizumab and Adalimumab

The study sought to evaluate the cost-effectiveness of Risankizumab compared to Adalimumab in chronic plaque psoriasis management, focusing on the differences in mean annual treatment expenses for both drugs. We used inverse probability treatment weighting to assess the uncertainties associated with the differences in cost and effectiveness, notably the average reduction in PASI scores. This accounted for factors such as patient age, gender, illness duration, duration of treatment, and number of comorbidities. A bootstrap approach with 10,000 replications was employed to generate 95% confidence intervals for the mean differences in cost and effectiveness. Four different scenarios were considered in the analysis to examine the cost-effectiveness of Risankizumab versus Adalimumab. The first scenario used the actual acquisition cost and dosages of both Risankizumab (i.e., 150 mg subcutaneously at week 0, week 4, and every 12 weeks after that) and Adalimumab (i.e., 80 mg subcutaneously once, then, after 1 week, 40 mg subcutaneously every other week). Second scenario assumed weekly dosing of Adalimumab (i.e., 80 mg subcutaneously once, then, after 1 week, 40 mg subcutaneously every week) since some patients who failed to have meaningful reductions in BSA (%) or PASI scores after a reasonable treatment duration (i.e., 24 weeks) with the standard dose every other week, respond better to every week dose of Adalimumab and achieve PASI 75 [34, 35]. The third scenario assumed weekly dosing of Adalimumab like the second scenario, but used the cheapest available biosimilar version of Adalimumab rather than the bio-originator with an assumption of similar clinical outcomes. The fourth scenario postulated a weekly dose of Adalimumab, the most affordable biosimilar Adalimumab, and a 40% discounted price of Risankizumab. This concession that could potentially be part of a confidential agreement with the drug manufacturer for the public health sector. Costs were expressed in United States Dollars (USD). All statistical analyses were performed using SAS® version 9.4 (SAS® Institute, Cary, NC, USA).

## Results

### Patients’ baseline characteristics

The analysis included 30 patients treated with Risankizumab and 40 patients with Adalimumab, all fulfilling the inclusion criteria. The average age of these patients was around 36 years, and there was no substantial age difference between those treated with Risankizumab and those treated with Adalimumab (39.43 years vs. 32.83 years, *p*-value = 0.0601). The majority of the studied population were males (64.29%) and, the gender distribution among those treated with either Risankizumab or Adalimumab showed no significant differences (*p*-value = 0.0612). The mean duration of the disease for patients treated with Risankizumab was almost 7 years compared to nearly 11 years among those treated on Adalimumab. However, this discrepancy was not statistically significant (*p*-value = 0.2274). Notably, the duration of therapy was significantly shorter for Risankizumab-treated patients compared to those treated with Adalimumab (11.12 months vs. 26.4 months, *p*-value < 0.0001).

The mean body weight and height were 86 kg and 167 cm, respectively, with no significant differences between patients treated with Risankizumab and their counterparts on Adalimumab. Most patients were otherwise healthy with patients on Risankizumab seemingly having higher mean number of comorbidities than their counterparts on Adalimumab (1.13 vs. 0.68, *p*-value = 0.2173). The mean percentage of BSA affected by plaque psoriasis at baseline was almost identical in patients treated with Risankizumab and those treated with Adalimumab (22.71% vs. 23.62%, *p*-value = 0.8842). On the other hand, the mean PASI score at baseline for patients treated with Risankizumab was higher than those treated with Adalimumab (27.78 vs. 17.91, *p*-value = 0.1109), but this difference was not statistically significant. A summary of these baseline characteristics can be found in Table 1. Significant reductions in the PASI scores and BSA were observed, with patients on Risankizumab demonstrating greater reductions in both PASI scores (-23.54 vs. -13.71) and BSA (-21.57 vs. -18.41) compared to their counterparts on Adalimumab as shown in Table 2.

**Table 1:** Patients’ baseline characteristics

Characteristic		Risankizumab (n = 30)	Adalimumab (n = 40)	*p*-value	Total
Gender	Male, n (%)	23(76.67)	22(55.00)	0.0612	45(64.29)
	Female, n (%)	7(23.33)	18(45.00)		25(35.71)
Age (yrs.), mean ± SD		39.43 ± 15.77	32.83 ± 13.12	0.0601	35.66 ± 14.58
Duration of illness (yrs.), mean ± SD		6.95 ± 7.97	10.50 ± 9.47	0.2274	9.13 ± 8.19
Duration of therapy (months), mean ± SD		11.12 ± 5.53	26.4 ± 18.66	< 0.0001	19.85 ± 16.37
Weight (KG), mean ± SD		93.53 ± 32.63	80.67 ± 23.20	0.0719	86.18 ± 28.16
Height (cm), mean ± SD		168.13 ± 10.02	165.91 ± 9.51	0.3556	166.85 ± 9.72
Body Surface Area (BSA), mean ± SD		22.71 ± 24.43	23.62 ± 23.59	0.8842	23.28 ± 23.72
PASI score, mean ± SD		24.78 ± 20.39	17.91 ± 12.65	0.1109	20.86 ± 16.65
Hypertension, n (%)		5(16.67)	4(10.00)	0.4831	9(12.86)
Diabetes, n (%)		5(16.67)	6(15.00)	1.00	11(15.71)
Asthma, n (%)		0(0.0)	2(5.00)	0.5031	2(2.86)
Cardiovascular disease (CVD), n (%)		2(6.67)	0(0.0)	0.1801	2(2.86)
Dyslipidemia, n (%)		7(23.33)	7(17.50)	0.5460	14(20.00)
Number of comorbidities, n (%)		1.13 ± 1.74	0.68 ± 1.16	0.2173	0.87 ± 1.44

**Table 2:** Difference in PASI scores and BSA at baseline and follow-up

PASI score				
Monoclonal antibody (mAb)	Baseline	Follow-up	Mean difference with 95% CI	*p*-value
Adalimumab	17.91 ± 12.65	4.19 ± 9.08	–13.71(–17.93 - − 9.49)	< 0.0001
Risankizumab	24.78 ± 20.39	1.25 ± 2.15	–23.54(–31.27 - − 15.80)	< 0.0001
**BSA (%)**				
Monoclonal antibody (mAb)	Baseline	Follow-up	Mean difference with 95% CI	*p*-value
Adalimumab	23.62 ± 23.59	5.21 ± 14.38	–18.41(–26.56 - − 10.27)	< 0.0001
Risankizumab	22.71 ± 24.43	1.14 ± 1.84	–21.57(–36.38 - − 12.06)	0.0005

### Regression models examining the impact of Risankizumab vs. Adalimumab on PASI scores and BSA

**Fig. 1:**
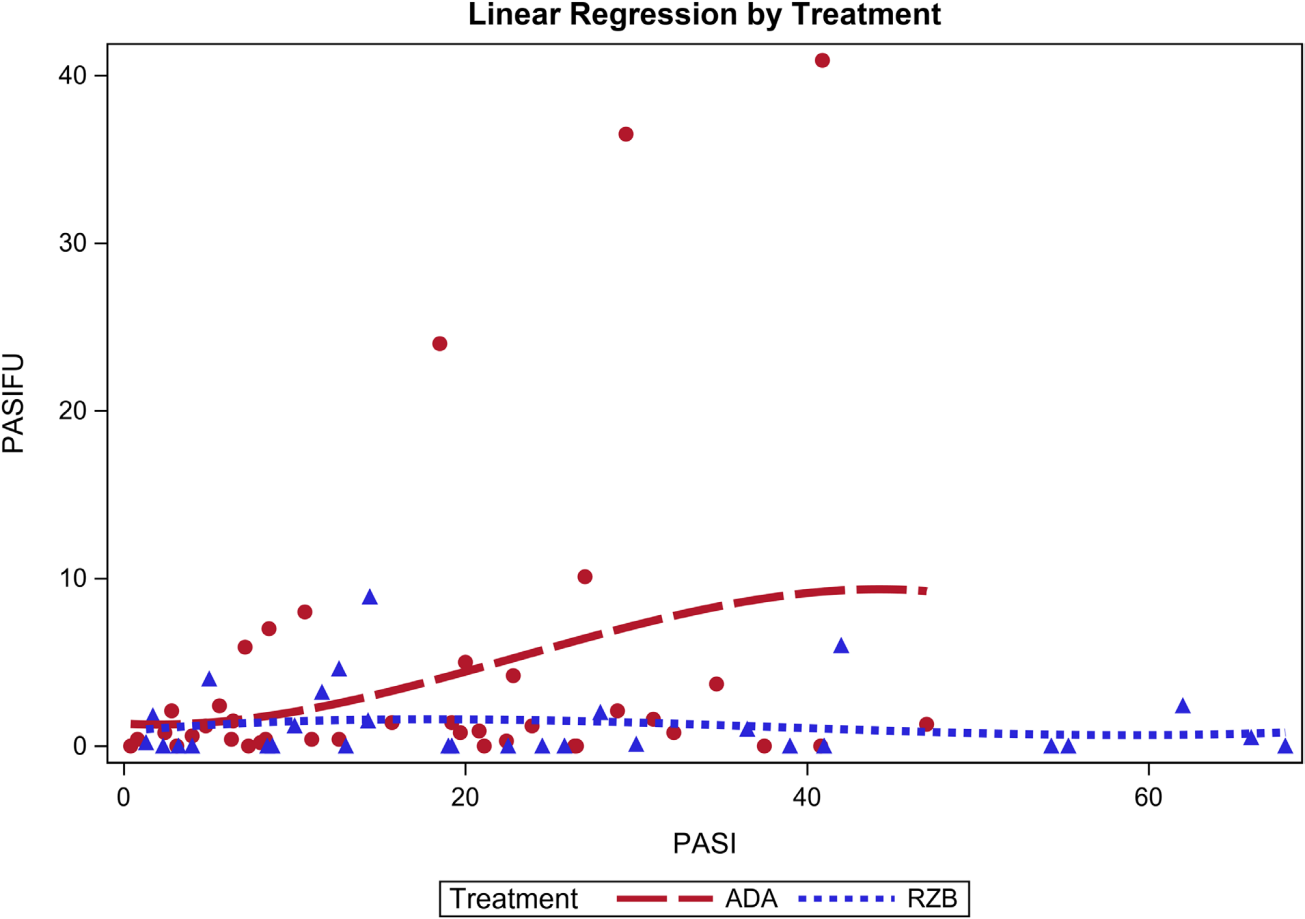
The relationship between baseline and follow-up PASI scores for Adalimumab (ADA) and Risankizumab (RZB)

Patients exhibiting a baseline PASI score > 10, indicative of severe disease, were more likely to have a PASI score at follow-up (PASIFU) lower than five, suggestive of mild disease, as shown by the regression line in Fig. 1. Despite the seeming superiority of Risankizumab in promoting a more substantial reduction in PASI score at follow-up compared to Adalimumab, the statistical significance of this observation was not confirmed (*p*-value = 0.1023). Risankizumab-treated patients exhibited higher odds of achieving PASI 75 in comparison to their counterparts on Adalimumab. However, after adjusting for variables such as age, gender, illness and treatment durations, and the number of comorbidities, this observation did not reach statistical significance (OR = 2.75, 95% CI = [0.667–11.14], *p*-value = 0.1628). This finding is detailed in Table 3.

Similarly, patients treated with Risankizumab were more likely to achieve a more substantial decrease in the affected BSA compared to their counterparts treated with Adalimumab, but again this was not statistically significant after controlling for age, gender, duration of illness, duration of treatment, and number of comorbidities (β = 34.11, 95% CI = [–14.926–83.166], *p*-value = 0.1687) as shown in Table 4.

**Table 3:** Analyzing the Association Between Risankizumab Use and Achieving PASI 75 in Psoriasis Patients through Multiple Logistic Regression

Variable	Odds ratio (OR)	*p*-value	95% Confidence Interval (CI)
Risankizumab vs. Adalimumab	2.725	0.1628	0.667–11.140
Age	0.966	0.2243	0.913–1.021
Female vs. male	0.695	0.5558	0.207–2.332
Duration of illness	0.999	0.9888	0.927–1.077
Duration of treatment	1.265	0.3665	0.759–2.109
Number of comorbidities	1.308	0.3797	0.719–2.381

**Table 4:** Assessing the Impact of Risankizumab on Body Surface Area Reduction in Psoriasis Patients: A Multiple Linear Regression Analysis

Variable	β-regression coefficient	*p*-value	95% Confidence Interval (CI)
Risankizumab vs. Adalimumab	34.119	0.1687	–14.926–83.166
Age	–1.408	0.1447	–3.314–0.499
Female vs. male	29.19	0.205	–16.438–74.819
Duration of illness	0.909	0.504	–1.801–3.619
Duration of treatment	–0.624	0.937	–16.382–15.135
Number of comorbidities	2.197	0.804	–15.441–19.836

### Cost effectiveness of Risankizumab *versus* Adalimumab in reducing PASI scores

**Fig. 2:**
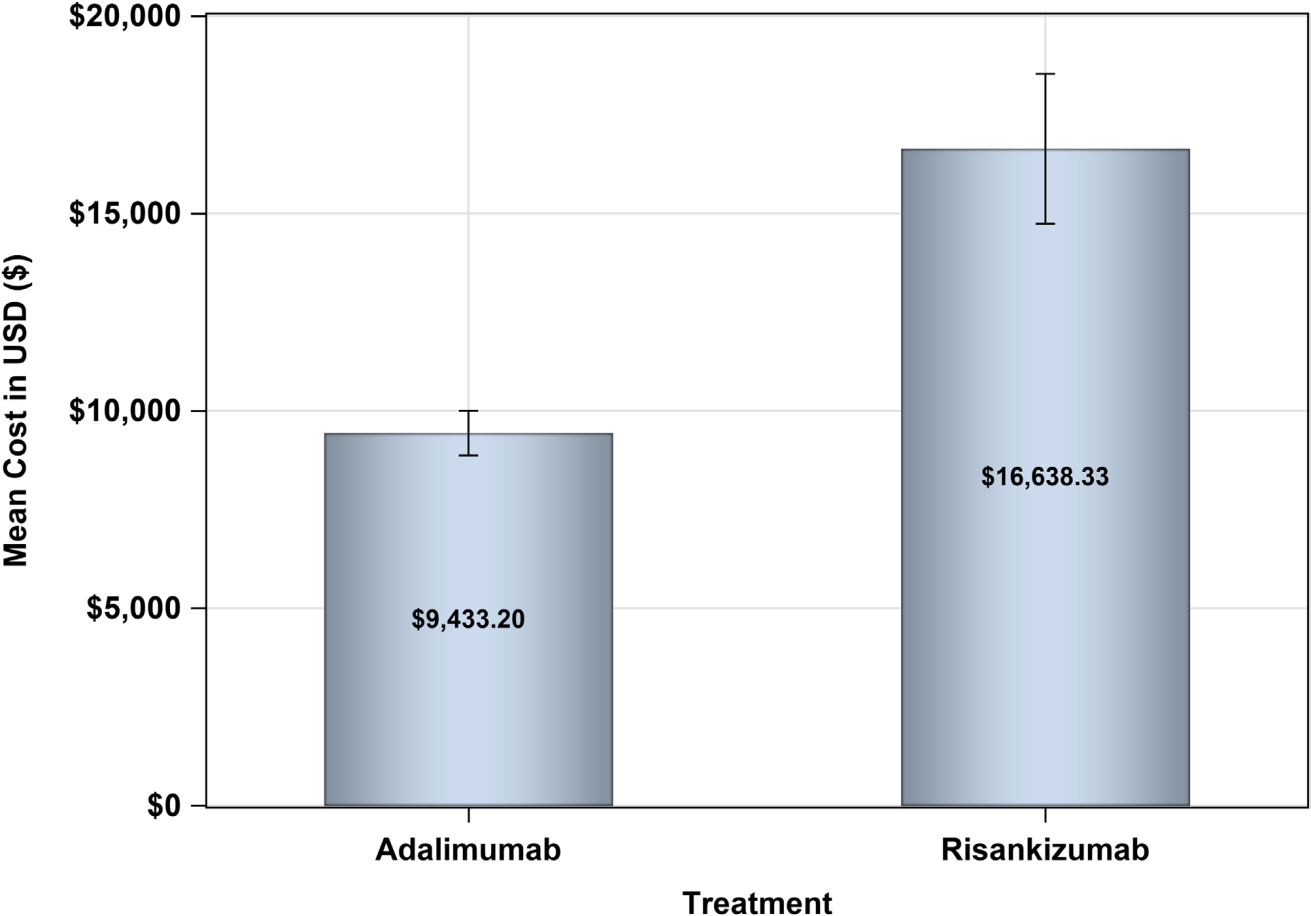
Comparative Annual Treatment Costs of Adalimumab vs. Risankizumab for Plaque Psoriasis: A Perspective from Saudi Arabia’s Public Health Sector

**Fig. 3:**
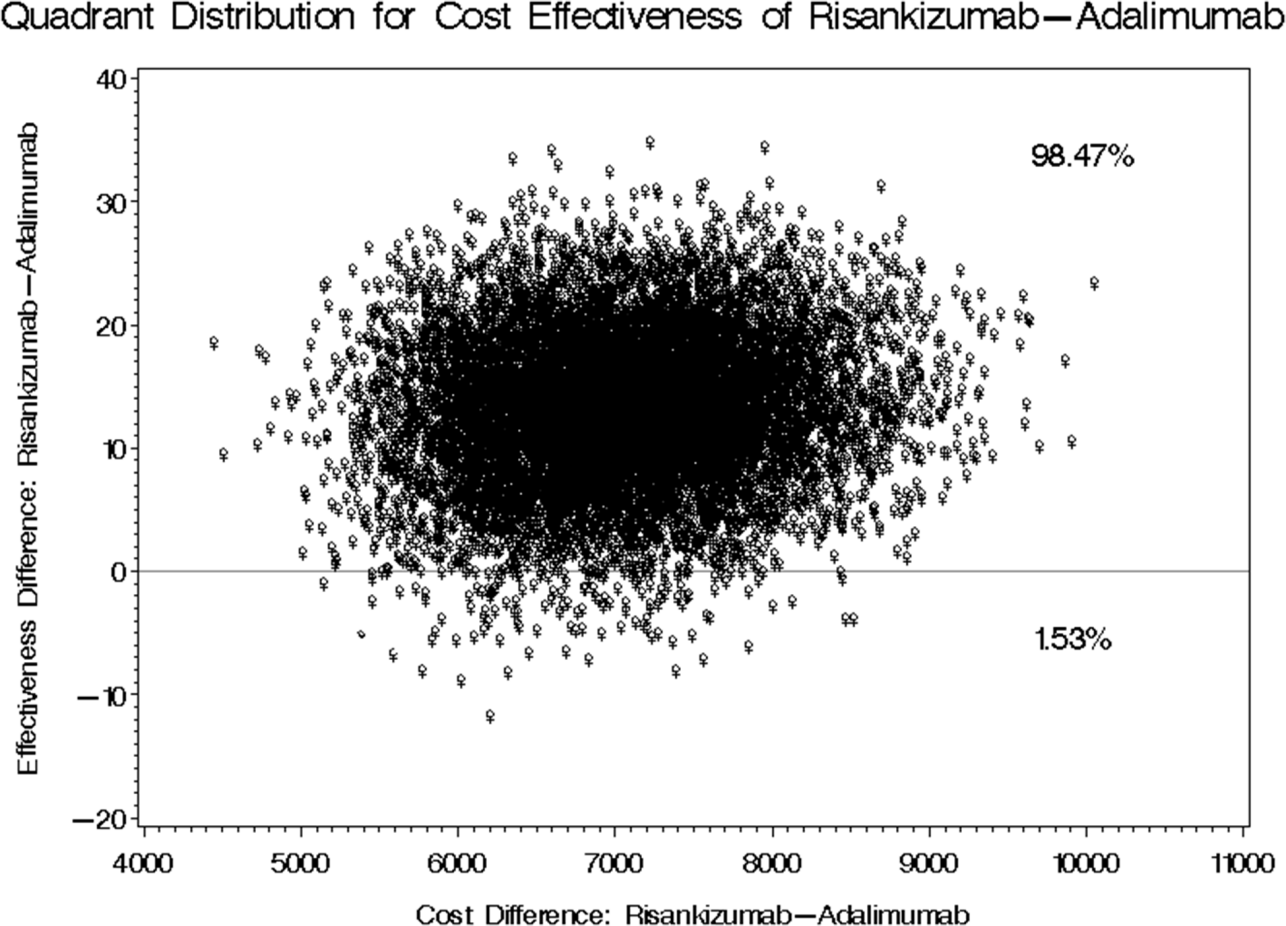
Bootstrap Distribution of Cost-Effectiveness: Comparative Analysis between Risankizumab and Adalimumab Incorporating Real Utilization Rates and Costs

The mean annual treatment cost for Risankizumab stood at USD 16,638.33 compared to USD 9,433.20 for Adalimumab, yielding a mean difference of USD 7,205.13 as shown in Fig. 2. The 95% bootstrap confidence interval for this annual cost difference ranged from USD 5,981.39 to USD 9,063.56. Regarding the mean difference in PASI score reduction, patients treated with Risankizumab outperformed those treated with Adalimumab by 12.88 (favoring Risankizumab). The 95% bootstrap confidence interval for this difference spanned from 1.271 to 24.934, as shown in Table 5, considering the first scenario of real acquisition cost of both treatments. Two cost-effectiveness quadrants were generated in this first scenario, where Risankizumab use led to higher costs but greater reductions in PASI scores 98.47% of the time. In comparison, higher costs with lower reductions were observed in 1.53% of cases (Fig. 3).

**Table 5:** Comparison of Mean PASI Score and BSA Reductions, along with Treatment Costs, in Psoriasis Patients Treated with Risankizumab (N = 30) vs. Adalimumab (N = 40)

	Risankizumab	Adalimumab	Mean difference (95% confidence interval)
Cost of treatment (USD), mean ± SD	16638.33 ± 5086.63	9433.20 ± 1768.37	7,205.13(5981.39–9063.56)
Difference in PASI (%)	87.373 ± 25.938	74.499 ± 36.143	12.88(1.271–24.934)

**Fig. 4:**
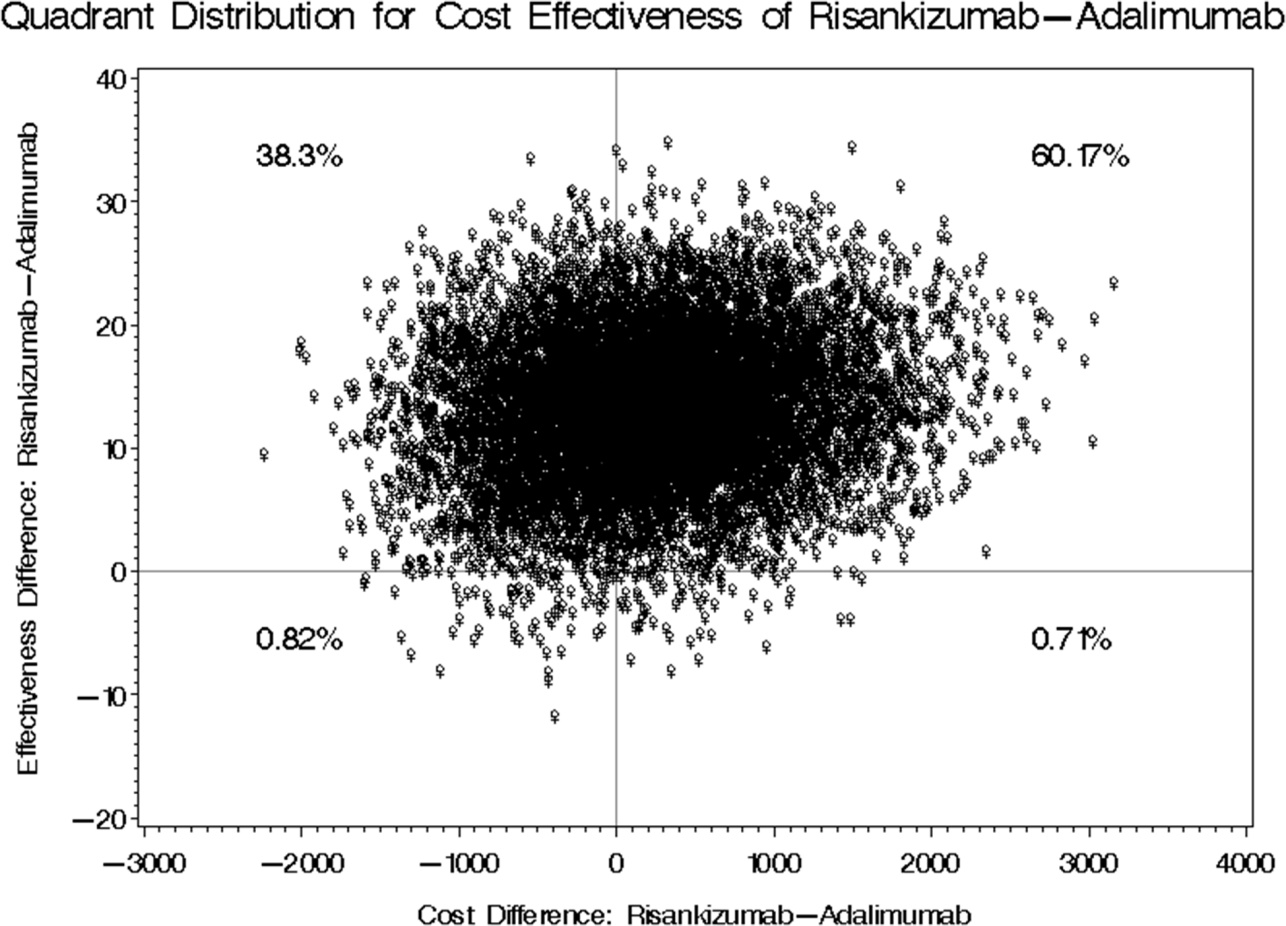
Bootstrap Distribution of Cost-Effectiveness: Risankizumab vs. Adalimumab under Weekly Dosing Assumption for Adalimumab

In the second scenario which used the real effectiveness outcome (i.e., PASI score reduction) but assumed double dosing of Adalimumab (i.e., every week dosing instead of two weeks), the mean annual difference between Risankizumab and Adalimumab was USD 517.81 with bootstrap 95% confidence interval ranging from USD − 567.699 to 2,525.28 as shown in Table 6. Four cost effectiveness quadrants were generated for the second scenario. Risankizumab resulted in higher cost and greater reduction in PASI scores in 60.17% of the bootstrap distributions, higher cost and lower reduction in PASI scores in 0.71% of the bootstrap distributions, lower cost and greater reduction in PASI scores in 38.3% of the bootstrap distributions, and lower cost and lower reduction in PASI scores in 0.82% of the bootstrap distributions as shown in Fig. 4.

**Table 6:** Weekly Dosing Assumption for Adalimumab: Comparison of Mean PASI Score, BSA Reduction, and Treatment Cost in Psoriasis Patients on Risankizumab (N = 30) vs. Adalimumab (N = 40)

	Risankizumab	Adalimumab	Mean difference (95% confidence interval)
Cost of treatment (USD), mean ± SD	16638.33 ± 5086.63	16120.52 ± 1039.43	517.81(–567.699–2525.28)
Difference in PASI (%)	87.373 ± 25.938	74.499 ± 36.143	12.88(1.271–24.934)

**Fig. 5:**
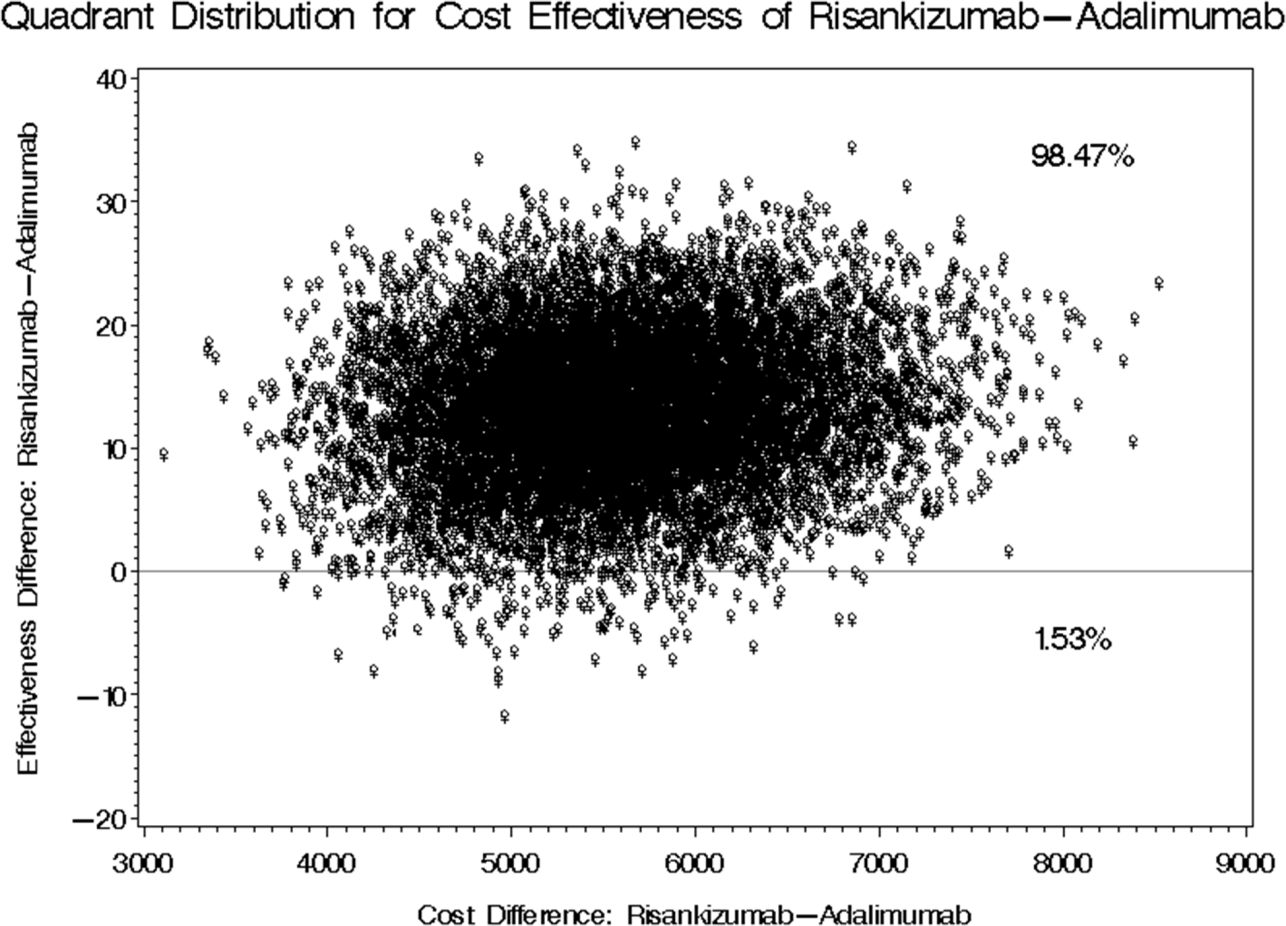
Bootstrap distribution of cost-effectiveness for the Risankizumab versus Adalimumab assuming double dosing and lowest price for Adalimumab assumption

The third scenario assumed no change in the effectiveness outcome (i.e., PASI score reduction) but incorporated double Adalimumab dosing and use of the least costly Adalimumab biosimilar. The mean annual cost difference between Risankizumab and Adalimumab stood at USD 5,870.93, with the 95% bootstrap confidence interval spanning from USD 4,772.39 to USD 7,865.18 (Table 7). This scenario mirrored the first, generating two cost-effectiveness quadrants wherein Risankizumab led to higher costs with either greater or lower reductions in PASI scores in 98.47% and 1.53% of cases, respectively (Fig. 5).

**Fig. 6:**
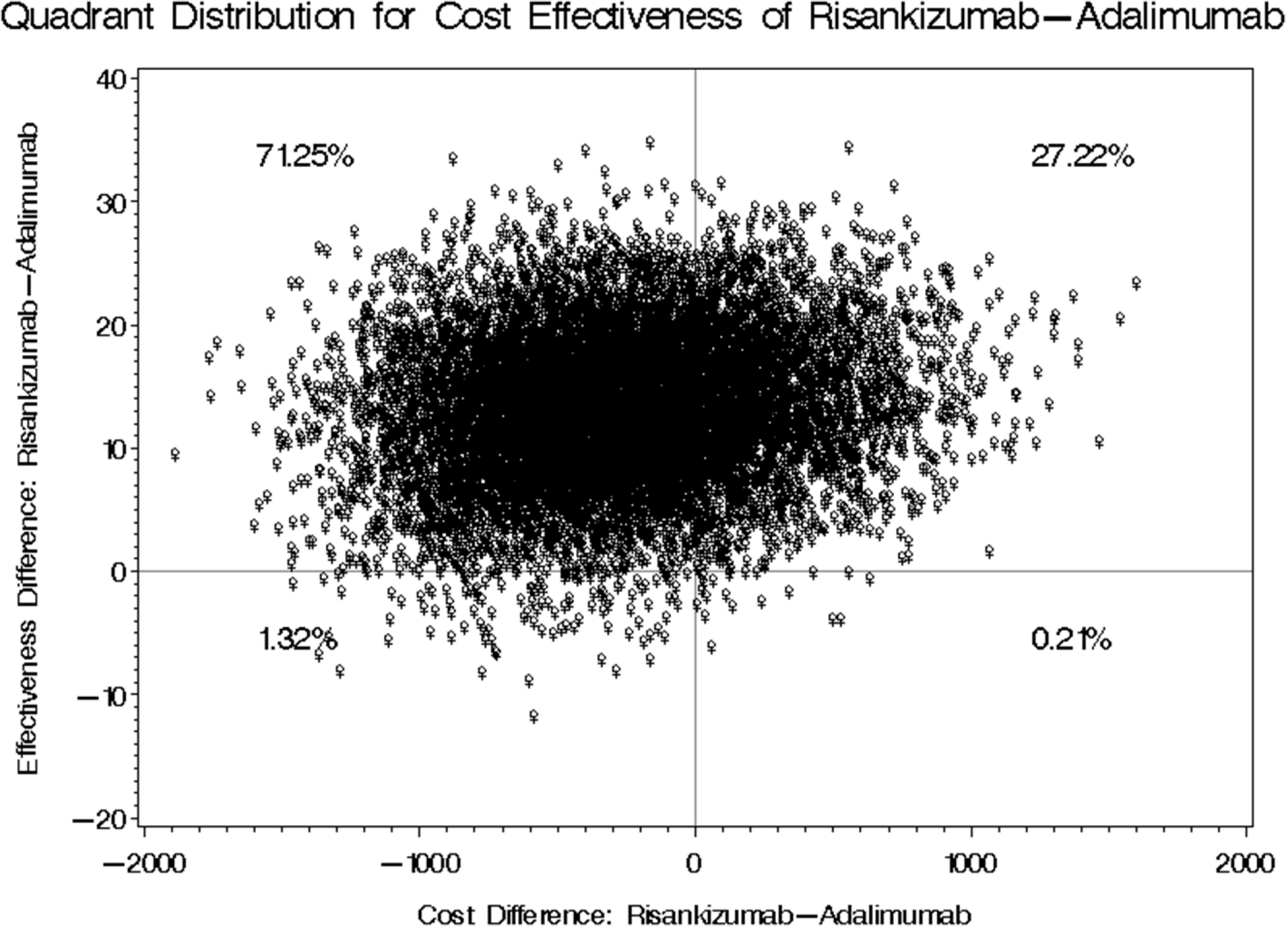
Bootstrap Distribution of Cost-Effectiveness: Risankizumab vs. Adalimumab, Assuming Weekly Dosing, Cheapest Biosimilar for Adalimumab, and a 40% Public Price Reduction for Risankizumab

The fourth scenario, which also maintained consistent effectiveness outcomes but assumed double Adalimumab dosing (i.e., every week dosing instead of two weeks), the use of a biosimilar version of Adalimumab with the lowest acquisition cost, and 40% discounted acquisition price of Risankizumab for public health sector, the mean difference of annual treatment cost between Risankizumab and Adalimumab was USD 20.01 with bootstrap 95% confidence interval ranging from USD − 1140.65 to USD 683.03 as shown in Table 8. Four cost effectiveness quadrants were generated for the fourth scenario. Risankizumab resulted in higher cost and greater reduction in PASI scores in only 27.22% of the bootstrap distributions, higher cost and lower reduction in PASI scores in 0.21% of the bootstrap distributions, lower cost and greater reduction in PASI scores in 71.25% of the bootstrap distributions, and lower cost and lower reduction in PASI scores in 1.32% of the bootstrap distributions as shown in Fig. 6.

**Table 7:** Assuming Weekly Dosing and Cheapest Biosimilar for Adalimumab: Comparison of Mean PASI Score, BSA Reduction, and Treatment Cost in Psoriasis Patients on Risankizumab (N = 30) vs. Adalimumab (N = 40)

	Risankizumab	Adalimumab	Mean difference (95% confidence interval)
Cost of treatment (USD), mean ± SD	16638.33 ± 5086.63	10767.40 ± 1008.35	5870.93(4772.39– 7865.18)
Difference in PASI (%)	87.373 ± 25.938	74.499 ± 36.143	12.88(1.271–24.934)

**Table 8:** Comparing Mean PASI Score, BSA Reduction, and Treatment Cost: Assumptions of Weekly Dosing and Cheapest Biosimilar for Adalimumab with a 40% Risankizumab Price Discount (Risankizumab N = 30 vs. Adalimumab N = 40)

	Risankizumab	Adalimumab	Mean difference (95% confidence interval)
Cost of treatment (USD), mean ± SD	10787.41 ± 3170.08	10767.40 ± 1008.35	20.01(-1140.65–683.031)
Difference in PASI (%)	87.373 ± 25.938	74.499 ± 36.143	12.88(1.271–24.934)

## Discussion

The use of Risankizumab for the management of plaque psoriasis has been advocated by some local medical experts over older, cheaper, and commonly utilized biologics, such as Adalimumab [31]. Several key findings need to be discussed in this study that evaluated the clinical effectiveness and direct medical costs of Risankizumab versus Adalimumab in Saudi Arabia. Risankizumab treatment resulted in larger reductions in both PASI scores and BSA compared to Adalimumab despite the shorter duration of therapy for patients treated with Risankizumab compared to their counterparts on Adalimumab. However, the likelihood of achieving PASI 75 or greater reduction in the affected BSA among patients treated with Risankizumab compared to their counterparts on Adalimumab was not statistically significant. On the other hand, the mean annual treatment cost for Risankizumab was higher than Adalimumab, despite the former leading to a more significant reduction in PASI scores. Therefore, this study assessed the incremental effectiveness of Risankizumab versus Adalimumab using four scenarios based on observed clinical practice. In two of these scenarios where the use of Risankizumab for the treatment of plaque psoriasis was compared to weekly dosing of Adalimumab, Risankizumab was found to be either cost-saving or resulted in negligible incremental cost but with greater effectiveness especially, when discounts on the acquisition price of Risankizumab were considered.

These findings are in line with the findings of a network meta-analysis that compared Risankizumab to other biologics, such as tildrakizumab, ustekinumab, and TNF-inhibitors, and found Risankizumab to be more efficacious at inducing all levels of PASI response [21]. Additionally, Risankizumab was more efficacious than Adalimumab in a randomized, double-blind, active-control trial that compared both safety and efficacy of Risankizumab versus Adalimumab at 66 clinics in 11 countries among patients with moderate to severe plaque psoriasis [24]. However, these studies have either compared Risankizumab to Adalimumab indirectly or were conducted in controlled environments [21, 24]. With regard to the cost-effectiveness of Risankizumab, it was projected based on a Markov cohort-level model that Risankizumab will lead to 0.3–0.89 quality-adjusted life years (QALY) versus other approved biologics for plaque psoriasis in Japan. However, this comes with an incremental cost ranging from approximately USD 17,000 per QALY when compared to Ustekinumab to USD 41,000 per QALY when compared to Adalimumab, which is deemed to be cost-effective from both the healthcare payer and societal perspectives in Japan [36]. In contrast with the findings of the economic evaluation of Risankizumab for plaque psoriasis in Japan [36], our study presents real-world clinical and economic evaluation from the perspective of the healthcare system in Saudi Arabia. Moreover, it presents the clinical effectiveness and direct medical costs associated with Risankizumab and Adalimumab considering different scenarios. These findings are also in line with another cost-effectiveness model conducted from the perspective of the National Health Service in the United Kingdom to determine the optimal place of biologic therapies for the management of psoriasis [37]. In this study that used a cohort-based Markov model, Adalimumab biosimilar was deemed to be the most cost-effective first-line treatment for psoriasis in the United Kingdom despite its lower likelihood to achieve PASI 75 and 90 when compared to Risankizumab [37].

Therefore, the findings of this study should encourage efficient utilization of resources for the management of psoriasis in Saudi Arabia at a time of increased awareness of the importance of spending efficiency in healthcare and the immense transformation in the public healthcare sector [38, 39]. The optimal utilization of Risankizumab as a second-line for plaque psoriasis patients who failed to achieve PASI 75 after being treated with Adalimumab every other week for a reasonable duration of time could be the efficient and optimal option in light of the findings of this study as well as other studies [37].

### Limitations of the study

Although this is the first study that examined the cost-effectiveness of Risankizumab versus Adalimumab using real-world data in Saudi Arabia, it has several limitations that must be acknowledged. The potential for information bias cannot be dismissed as data were gathered from electronic medical records (EMRs). Furthermore, unlike many health economic evaluations of psoriasis treatments, our study did not incorporate quality-of-life utility estimates like Quality-Adjusted Life Years (QALY). This omission is due to the absence of validated utility estimates applicable to the Saudi population [38]. We also did not evaluate the cost-effectiveness of using Risankizumab based on a cost-effectiveness threshold due to the absence of utility estimates (such as QALY) and a nationally recognized cost-effectiveness threshold in Saudi Arabia, despite recent research efforts [40]. Despite these limitations, the findings of this research should offer valuable insights for policymakers. This study illuminates the potential benefits of innovative therapies like Risankizumab, which, when utilized efficiently, can present cost savings and improved efficacy in treating plaque psoriasis.

## Conclusions

In conclusion, the employment of Risankizumab as a treatment for plaque psoriasis in Saudi Arabia could provide incremental effectiveness compared to Adalimumab, albeit at a higher acquisition cost. However, different scenarios, such as an increased dosage of Adalimumab or a biosimilar version of Adalimumab coupled with a discounted price for Risankizumab, can make Risankizumab cost-saving in a significant proportion of cases. Thus, the efficient allocation of resources in Saudi Arabia’s public health sector could enhance access to more effective biologic-based therapies like Risankizumab. The findings underscore the need for comprehensive healthcare reforms, including robust digital information systems and an emphasis on patient-centered care. Despite potential limitations like information bias and the lack of nationally recognized cost-effectiveness thresholds, this study offers valuable insights into improving the treatment of plaque psoriasis, thereby aiding policymakers in their healthcare reform and resource allocation decisions.

## Abbreviations

PASI: Psoriasis Area and Severity Index
BSA: Body surface area
PGA: Physician Global Assessment
DLQI: Dermatology Life Quality Index
FDA: Food and Drug Administration
RCTs: Randomized controlled trials
NMA: Network meta-analysis
QoL: Quality of life
mAbs: Monoclonal antibodies
USD: United States Dollars
MENA: Middle East and North Africa
MOH: Ministry of Health
AWP: Average wholesale prices
QALY: Quality-adjusted life years
